# Scoliosis epidemiology is not similar all over the world: a study from a scoliosis school screening on Chongming Island (China)

**DOI:** 10.1186/s12891-016-1140-6

**Published:** 2016-07-22

**Authors:** Qing Du, Xuan Zhou, Stefano Negrini, Nan Chen, Xiaoyan Yang, Juping Liang, Kun Sun

**Affiliations:** Department of Rehabilitation Medicine, Xin Hua Hospital Affiliated to Shanghai Jiao Tong University School of Medicine, Shanghai, 200092 China; Department of Clinical and Experimental Sciences, University of Brescia, Brescia, Italy; IRCCS Fondazione Don Gnocchi, Milan, 20141 Italy; Department of Pediatric cardiology, Xin Hua Hospital Affiliated to Shanghai Jiao Tong University School of Medicine, Shanghai, 200092 China

**Keywords:** School scoliosis screening, Scoliosis, Prevalence, China

## Abstract

**Background:**

School scoliosis screening has been carried out around the world. The screen program has never been performed on Chongming Island, the third largest island in China and characterized less population exchange with the rest of China. This study was designed to examine scoliotic parameters in children from Chongming Island and determine whether the parameters differed from those of the published data.

**Methods:**

A total of 6824 children (3477 boys and 3347 girls) aged from 6 to 17 were recruited. The screen included Adam’s test and scoliometer measurements. Posteroanterior radiographic evaluation was performed if trunk rotation was 5° or more.

**Results:**

One hundred seventy two were confirmed with Cobb angle of 10° or more; the prevalence was 2.52 %, higher in girls (3.11 %) than in boys (1.96 %) (*p* < 0.05). There was a weak positive correlation between prevalence and age. Majority curves were minor (from 10 to 19°). The most common thoracic curve was right curve (60.3 % of all thoracic curves), while the most common thoracolumbar (75.5 %) and lumbar curves (64.7 %) were left curves.

**Conclusions:**

The prevalence of scoliosis on Chongming Island was 2.52 %. This study indicates that epidemiology of scoliosis has regional variation, and genetic differences may contribute such difference.

**Electronic supplementary material:**

The online version of this article (doi:10.1186/s12891-016-1140-6) contains supplementary material, which is available to authorized users.

## Background

Scoliosis is usually detected through a school screening program. School scoliosis screening (SSS) is considered a powerful tool that can identify individuals with unrecognized scoliosis at an early stage when less invasive treatment is more effective [1]. SSS can also generate invaluable data regarding not only the prevalence but also the natural history of scoliosis, which contribute significantly to the research for idiopathic scoliosis etiology [2]. The progression of untreated idiopathic scoliosis in patients during periods of rapid growth may result in severe deformity, which may be accompanied by restrictive pulmonary disease [3–5], and social psychogenic problems [6, 7]. Early detection by screening programs and application of an effective orthopedic and rehabilitation treatment are essential for avoiding scoliosis progression, minimizing the need for an operation [8–13], and reducing associated costs [14–16].

SSS is conducted around the world [2]. In Europe, SSS dates back to the early 20th century. In the USA, SSS has been conducted since the early 1960s; currently, less than half of the states have legislated school screening. In Japan, school screening programs for scoliosis are mandatory by law. In the past, SSS was performed in many countries. In China, SSS was not performed until 1985. As of 2012, only three provinces and one municipality in China had conducted SSS [17–21]. At present, a SSS program is not included in the Chinese School Health Service, and there is no recommendation to include one. There are no national screening programs in China: the only established SSS program is in Hong Kong. To date, SSS has never been performed on Chongming Island, which is the third largest island in China, with a population of 703,722.

Although the pathogenesis of idiopathic scoliosis remains controversial, genetic factors are thought to play key roles in the development of idiopathic scoliosis etiology [22]. Genetic factors in China could be different than in other places and may be reflected in differences in prevalence. Race may influence an individual’s natural spinopelvic alignment [23]. The prevalence of scoliosis differs among races [24, 25], and curve severity is associated with race [25, 26]. Kebaish et al. [25] found that the prevalence of scoliosis was 11.1 % for whites and 6.5 % for African Americans. Zavatsky et al. [26] found that curve magnitude was greater in black patients than in white patients (33° vs. 28°). A study found that the prevalence of scoliosis in Chinese girls was significantly higher than in Malay and Indian girls aged 11 to 12 and 16 to 17 years [24]. Therefore, further studies are necessary to confirm the prevalence and distribution of various scoliotic parameters according to patient age and gender in a Chinese population.

The aim of the present study was to examine scoliotic parameters (such as patients’ age, gender, curve magnitude, curve type, and curve side) in children from Chongming Island, an area with few population exchanges with the rest of China, through a scoliosis school screening program and to determine whether the parameters differed from that of the previously published data.

## Methods

### Preliminary work

After permission was obtained from the Chongming Ministries of Education and Health, each school was contacted. It was essential to obtain the support of school physicians and the collaboration of the teachers and parents. Two months before starting the program, a member of the screening team gave a detailed explanation of the aims, importance, methods, and procedure of SSS to school physicians and teachers. Information leaflets were sent to all schools on Chongming Island, as well as to local education and health authorities, teachers, and parents. The parents were informed by means of a letter that described the intentions of the study, the clinical importance of early detection, and the details of the examination procedure. Informed consents were obtained from the parents of the students participating in the study.

Once this phase was completed, four scoliosis screening teams, consisting of a senior physician, a resident, a nurse, and a medical student, were organized and trained in screening methods and childhood behavior. By alternating the screening teams, fatiguing the medical staff was avoided. Before the screening, the forms were forwarded to the schools and the children were instructed to fill in their biographical information.

### School screening

The screening took place during regular lesson time to avoid any psychological stress, usually with the assistance of the school physician. Small groups of no more than ten children were admitted into the screening room at a time. Each child brought his or her completed data form. The boys and girls were examined separately, and each patient was screened in private. The children wore only a slip or undershorts for the examination.

The medical student recorded the children’s physical attributes. The child then proceeded to the resident or the senior physician, who checked for spinal and other deformities. A standard clinical examination preceded the screening tests. The screening examination began with the child standing up straight with their back to the examiner, head up and the arms relaxed at the sides. With the child in this position, we looked for shoulder asymmetries, scapular prominence, unequal waistline, and lower limb length inequality. Any abnormality of the torso or the lower extremities was recorded. The Adam’s forward bending test was then performed to check for signs of vertebral rotation and lateral deviation of the spine.

The parameters that were recorded included biographical information (name, date of birth, and family address), physical attributes (age of menarche for the girls, patient weight, patient height when standing), and abnormalities involving the trunk or spine (e.g., humps in the rib or lumbar region, discrepancies between the shoulders or between the hips, and imbalance of the torso or spine).

### Rescreening and radiographic evaluation

Healthy children exhibit symmetric shoulder levels, scapular prominence and waistline, equal length of lower limbs, and no vertebral rotation and lateral deviation of the spine. The asymmetry of the shoulder levels, scapular prominence and waistline, lower limb length inequality, and vertebral rotation and lateral deviation of the spine were recorded as absent or present. If any abnormality of all these signs was noted, it was considered positive sign. Children who showed at least one positive sign were re-examined by a senior physician to confirm the criteria for referral. Children in whom scoliosis was suspected were requested for re-examination, at which time a second Adam’s forward bending test was performed. The examiner compared the two sides of the torso at the thoracic, thoracolumbar, and lumbar levels. If any difference in height was noted, the angle of trunk rotation (ATR) was measured with a scoliometer, which is currently the best tool available for scoliosis screening. [10] A difference of five degrees or more was considered to be a positive finding on the bending test.

The children were referred for radiographic evaluation only if the examiner confirmed a positive result on the bending test. Posteroanterior radiographs were made (with the patient standing) at a local hospital or at Xin Hua hospital, which is affiliated with Shanghai Jiao Tong University School of Medicine. The curve magnitude was immediately measured in the coronal plane using the Cobb method. A curve of 10° or more was defined as scoliosis, as specified by the Scoliosis Research Society (SRS) and the International Scientific Society on Scoliosis Orthopaedic and Rehabilitation Treatment (SOSORT) guidelines [27]. The Cobb angle, curve type, and curve side of the scoliosis were recorded.

### Screening population

In our screening program, we included primary, junior high and senior high school children (aged 6–17 years), excluding grade three of senior high school. During the 2012 and 2013 academic years, 32,835 students (16,727 boys and 16,108 girls) aged 6 to 17 years were registered by the Educational Authorities on Chongming Island. There were a total of 76 schools on Chongming Island, including 40 primary schools, 34 junior high schools and 8 senior high schools.

In the present study, class was taken as a unit for cluster random sampling. A serial number was given to each class in each grade at each school. The class was then screened according to the random number created by the computer. We randomly sampled one class from each grade at each school. We planned to sample 40 classes from grades one through five in primary schools, 34 classes from grades one through four in junior high schools, and 8 classes from grades one to two in senior high schools. Three hundred fifty-two classes were randomly sampled from each grade of 40 primary schools (5 grades), 34 junior high schools (4 grades) and 8 senior high schools (2 grades). Exclusion criteria for classes included 1 or more children in a class with an ATR of 5° not having an X-ray taken, and the X-ray results of 1 or more children in a class not being collected. This was due to parents either refusing to have X-rays performed on their child or taking their child elsewhere to take a radiograph. In the end, 247 classes were included and 105 were excluded. There were 3477 boys and 3347 girls in the included population, while there were 1517 boys and 1391 girls in the excluded population. An ATR of 5° or more was measured in 442 members of the included population and 171 of the excluded population. There was no significant difference in gender and the rate of children with an ATR of 5° or more between the included and excluded populations. The loss rate was 29.9 %.

A total of 6824 children aged 6 to 17 years were screened from April through November 2012, which corresponds to 20.8 % of the relevant population on Chongming Island.

### Analysis of data

All parameters were recorded on a computer using Epidata 2.0. To calculate the prevalence rates, a curve of 10° or more was used as a cutoff point. All analyses were conducted using SPSS v.17.0. Chi-squared testing was used to identify statistically significant differences in the prevalence, the distribution of curve parameters and the variables of age and gender. Logistic regression analysis was used to identify correlations between the prevalence of scoliosis and the variables of age and gender. Linear regression analysis was used to identify correlations between curve magnitude and age. An effect size greater than 0.8 was considered large, approximately 0.5 was considered moderate, and less than 0.2 was considered small [28]. All hypothesis testing was considered significant at *p* < 0.05.

## Results

**Fig. 1:**
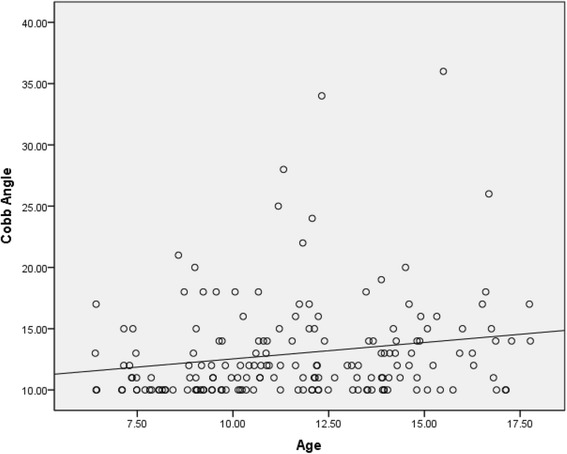
Correlation between age and Cobb angle

Out of the 6824 children in the screened population, 442 (201 boys and 241 girls) had clinical signs of scoliosis and were referred for radiological evaluation. A total of 172 children had radiographic evidence of scoliosis (a curve of 10° or more), demonstrating a prevalence rate of 2.52 % (Table 1). Logistic regression analysis showed that there was a significant association between the prevalence of scoliosis and the variables of age and gender. Linear regression analysis showed that there was a very weak, yet statistically significant, correlation between age and Cobb angle (Fig. 1). The correlation coefficient was 0.18 (*p* = 0.016).

**Table 1:** Clinical and radiologically detected scoliosis in children aged 6 to 17 years according to age and gender

Demographic variable	No. of children included (*N* = 6824)	No. of children referred for radiograph (*N* = 442)	No. of children in which scoliosis was radiologically detected (*N* = 172)	Prevalence of scoliosis (%)	Average Cobb angle (Degrees)
Age					
6~	163	15	4	2.45	12.5
7~	873	57	16	1.83	11.3
8~	853	39	12	1.41	12.1
9~	903	59	24	2.66	12.0
10~	849	60	23	2.71	12.4
11~	746	47	16	2.14	15.3
12~	592	44	17	2.87	13.7
13~	601	50	19	3.16	12.1
14~	529	29	18	3.40	13.1
15~	250	10	8	3.20	15.3
16~	247	18	10	4.05	15.1
17	218	14	5	2.29	13.0
Gender					
Boys	3477	201	68	1.96	12.5
Girls	3347	241	104	3.11	13.3

Of the 172 scoliosis patients (see Additional file 1), 68 were boys and 104 were girls. Girls had a significantly higher prevalence of scoliosis (3.11 %) than boys (1.96 %). Gender showed no statistically significant difference in the average Cobb angle. The male/female ratio was 1:1.5 overall but varied according to the magnitude of the curve (1:1.5 for curves of 10 to 19° and 1:2.3 for curves of 20 to 39°).

We found that 162 (94.2 %) of the scoliosis patients had mild curves (10 to 19°), and 10 (5.8 %) had moderate curves (20 to 39°). Most of the curves were small. There was no significant difference in the distribution of curve magnitude and age group. Gender also showed no statistically significant difference in curve magnitude: 95.6 % of boys (65/68) and 93.3 % of girls (97/104) had mild curves (Table 2).

**Table 2:** Curve magnitude by age and gender among the 172 patients with scoliosis

Demographic variable	No. of scoliosis patients with the following curve magnitudes	
	Cobb Angle (10 to 19°) (*N* = 162)	Moderate (20 to 39°) (*N* = 10)
Age		
6~	4	0
7~	16	0
8~	11	1
9~	23	1
10~	23	0
11~	13	3
12~	15	2
13~	19	0
14~	17	1
15~	7	1
16~	9	1
17	5	0
Gender		
Boys	65	3
Girls	97	7

In assessing the curve type among the 172 scoliosis patients, we found that 68 (39.5 %) had thoracic curves, 53 (30.8 %) had thoracolumbar curves, 34 (19.8 %) had lumbar curves, and 17 (9.9 %) had double curves. Thoracic curves were the most common type of curve identified, followed by thoracolumbar curves. Single curves occurred nine times more frequently than double curves. There was a statistically significant difference in the distribution of curve type and age group. Among age groups, most groups were more likely to have thoracic curves. However, the 6 year-old, 9 year-old and 11 year-old groups were more likely to have thoracolumbar curves, and the 12 year-old groups were more likely to have lumbar curves. Gender showed no statistically significant difference in curve type: boys and girls had statistically equivalent distributions of curve type (Table 3).

**Table 3:** Curve type by age, gender and curve side among the 172 patients with scoliosis

Demographic variable	Single curve			Double curve (*N* = 17)
	Thoracic (*N* = 68)	Thoracolumbar (*N* = 53)	Lumbar (*N* = 34)	
Age				
6~	1	3	0	0
7~	7	6	3	0
8~	8	3	1	0
9~	9	13	1	1
10~	8	5	5	5
11~	3	7	3	3
12~	4	5	6	2
13~	9	3	6	1
14~	9	5	2	2
15~	2	3	3	0
16~	4	0	3	3
17	4	0	1	0
Gender				
Boys	28	25	11	4
Girls	40	28	23	13
Side (major curve)				
Right	41	13	12	10
Left	27	40	22	7

Of the cases in the present study, thoracic curves were right in 60.3 %, thoracolumbar curves were left in 75.5 %, lumbar curves were left in 64.7 %, and major curves of double curves were right in 58.8 % (Table 3). There was a significant difference in the distribution of curve side and curve type.

There was a significant positive correlation between ATR and Cobb angle (*r* = 0.435, *P* = 0.000). ATR ranged from 5° to 18°, and Cobb angle ranged from 10° to 36°. Mean ATR was 5.8° ± 1.8°. Mean Cobb angle was 12.9° ± 4.1°. In thoracic curves, there was a significant positive correlation between ATR and Cobb angle (*r* = 0.256, *P* = 0.035). In thoracolumbar curves, there was a significant positive correlation between ATR and Cobb angle (*r* = 0.751, *P* = 0.000). In lumbar curves, there was a significant positive correlation between ATR and Cobb angle (*r* = 0.512, *P* = 0.002). There was no significant correlation between ATR and Cobb angle in double curves (*r* = -0.010, *P* = 0.969).

## Discussion

The prevalence rate of scoliosis in schoolchildren from Chongming Island aged 6 to 17 years was 2.52 %. There is an increase in the scoliosis prevalence of schoolchildren on Chongming Island compared to those reported by previous studies in other provinces of China (0.70 to 2.09 %) [17–21, 29], Singapore (0.38 to 1.2 %) [24, 30, 31], Japan (0.87 %) [32], Turkey (0.25 %) [16], Saudi Arabia (0.78 %) [33], India (0.13 %) [34], Minnesota (1.2 %) [9], Brazil (1.4–2.2 %) [35, 36], Greece (1.7 %) [37, 38], and Nigeria (1.2 %) [39]. The prevalence of scoliosis in our study is lower than those reported from Korea (3.26 %) [40], and Australia (3.1 %) [41]. In the Australian study [41], a different criterion (the presence of scoliosis was defined as a Cobb angle of 5° or more) was used. Similar criteria were used in the Korean study [40], but the screening population (children aged 10 to 14 years) was different from our population (children aged 6 to 17 years).

We found that there was a positive, but very weak, correlation between scoliosis and age. This finding is similar to that of reports from Singapore and Tokyo [30–32], but differs from that of the report from Korea [40]. Studies in Singapore found that there was an increasing trend in the prevalence of idiopathic adolescent scoliosis among female students between the ages of 9 and 14 years [30, 31]. Ueno et al. [32] reported that the prevalence of scoliosis in girls in Tokyo increased from 0.78 % at 11 to 12 years of age to 2.51 % at 13 to 14 years of age. Suh et al. [40] found that the prevalence of scoliosis in girls in Korea was higher at 10 to 12 years of age than at 13 to 14 years of age (5.57 % vs. 3.90 %). However, logistic regression analysis has never been used to identify correlations between the prevalence of scoliosis and age in all of these previous studies. Age group analysis showed no statistically significant difference in curve magnitude or curve side.

We also found that girls had a significantly higher prevalence of scoliosis than boys. This finding is in agreement with those described in previous studies [30, 32, 38] Gender was not associated with curve magnitude, curve type or curve side. The male/female ratio in the present study was 1:1.5, similar to that reported from the Shanxi Province of China (1:1.2) in 1995 [18]. In recent studies, Jenyo et al. [39] reported the highest male/female ratio (1:0.7) in Nigeria; however, the sample size was too small (a little more than 400) to be significant. Most studies reported a male/female ratio between 1:2.1 and 1:11.6 (1:2.1 for Greece, 1:2.4 for Korea, 1:2.6 for Turkey, 1:4.6 for Singapore, and 1:11.6 for Japan) [16, 30, 32, 38, 40], Based on recent publications, the male/female ratio in this study is higher than most. It seems that the proportion of male patients is higher in China.

The findings of the present study indicate that small scoliotic curves (10 to 19°) are the most common (94.2 %). This finding is in agreement with those described in reports from Korea, Tokyo, Turkey and India [16, 32, 34, 40].

The most common types of scoliotic curves in the present study were thoracic and thoracolumbar. This finding is in agreement with those described in reports from Brasil, Singapore, Nigeria and Crete [30, 35, 37, 39], but differs from those described in reports from Greece, where the proportion of single thoracolumbar and lumbar curves greatly outweighed that of all others [38]. In contrast, the prevalence of thoracic curves reported from Korea (47.6 % of 37,339 scoliotic curves) was greater than that reported in the present study (39.5 % of 172 scoliotic curves) [40]. In the present study, the distribution of curve type varied according to age. The finding that double curves constitute less than 10 % of the curves observed is in agreement with that reported from Korea [40].

In this study, most of thoracolumbar and lumbar curves were to the left. Thoracic curves were right in 60.3 %, thoracolumbar curves were left in 75.5 %, lumbar curves were left in 64.7 %, and major curves of double curves were right in 58.8 %. These findings differ from those of the epidemiological study from Greece [38]. Soucacos et al. [38] found that 75.5 % of thoracic curves were right, 66.3 % of thoracolumbar curves were left, and 76.2 % of lumbar curves were left. It seems that there are fewer right thoracic curves, more left thoracolumbar curves, and fewer left lumbar curves in Chinese scoliosis patients.

A positive correlation between ATR and Cobb angle was found in this study. The correlation coefficient between ATR and Cobb angle was consistent with the previous studies [42, 43]. The correlation between ATR and Cobb angle in thoracic curves was weaker than that reported by Carlson [44]. However, the curve magnitude in Carlson’s study (29°–79°) was larger than that in present study (10°–36°).

The percentage of schoolchildren referred for radiographs in the present study (6.5 %, 442 of 6824) is within the range reported from other countries (0.9 to 9.6 %) [9, 16, 18, 19, 37, 40]. In this study, we have reported a prevalence that we think is representative of the scoliotic population on Chongming Island and have further checked factors such as age, male/female ratio, curve magnitude, curve type, and curve side. Scoliosis is common in schoolchildren on Chongming Island and therefore poses a major public health problem. SSS appears to be an effective means for early detection (identification of a large number of previously undiagnosed curves), identification of children at a high risk for curve progression and selection of an effective orthopedic and rehabilitation treatment [9, 16, 37, 39]. The present study provides evidence to support the case for including SSS in annual school-based health screening programs in primary, junior and senior high schools on Chongming Island.

This study was not without limitations. The complete lost rate was 29.9 %: 3152 of 9976 children were excluded.

Additional investigations are underway to refine our understanding of scoliosis in the Chinese population: an epidemiological study of a larger population cohort and a follow-up study of the patients in our study who were identified as having scoliosis.

## Conclusions

We found that the overall prevalence of scoliosis was 2.52 % in Chongming Island. The percentages of curve magnitude and type were comparable. The following points in our study differed from the findings of research in other countries: (1) the proportion of male scoliosis patients was increased, (2) most of thoracolumbar and lumbar curves were to the left, and (3) the prevalence rate of scoliosis was associated with age. According to these results, epidemiological regional variability, possibly with genetic basis, should be considered.

All the characteristics we found may contribute to draw up health policies for the local government. They could help to develop a rehabilitation program for these patients in an early stage, and also improve their spinal health.

## Abbreviations

ATR, angle of trunk rotation; SOSORT, the International Scientific Society on Scoliosis Orthopaedic and Rehabilitation Treatment; SRS, scoliosis research society; SSS, school scoliosis screening

## Additional file

Additional file 1: The raw data of 172 scoliosis patients. (XLSX 23 kb)
